# Real‐world data of *Helicobacter pylori* prevalence, eradication regimens, and antibiotic resistance in Thailand, 2013–2018

**DOI:** 10.1002/jgh3.12208

**Published:** 2019-06-24

**Authors:** Rossanun Shoosanglertwijit, Nuttamon Kamrat, Duangporn Werawatganon, Tanittha Chatsuwan, Supakarn Chaithongrat, Rungsun Rerknimitr

**Affiliations:** ^1^ Department of Physiology, Faculty of Medicine Chulalongkorn University Bangkok Thailand; ^2^ Department of Microbiology, Faculty of Medicine Chulalongkorn University Bangkok Thailand; ^3^ Department of Internal Medicine, Faculty of Medicine Chulalongkorn University Bangkok Thailand

**Keywords:** antibiotic resistance, *Helicobacter pylori*, recommended regimen, treatment failure in Thailand

## Abstract

**Background and Aim:**

*Helicobacter pylori* is a class I carcinogen. Nowadays, the problem of antibiotic resistance is increasing worldwide. The latest prevalence rates of infection and resistant status in Thailand vary or are out of date. Our aims are to identify the current prevalence and antibiotic resistance patterns in Thailand and to suggest regimens for treatment‐naive and ‐resistant patients.

**Methods:**

This descriptive retrospective study was conducted, using a urea breath test, on patients in King Chulalongkorn Memorial Hospital between 2013 and 2017. They were categorized into the diagnostic group and posttreatment group. Specimens from some patients were cultured to identify the antibiotic‐resistant pattern.

**Results:**

There were 1894 patients included in our study. The prevalence of *H. pylori* infection in dyspeptic patients was 28.4%. Of 1258 patients, 1165 (92.61%) responded to initial treatment. The 95 patients who failed to respond could respond to second‐line treatment of longer period, at higher doses, or using other antibiotics (success rate 68.42%). There were 21.43, 14.29, and 10.71% of patients resistant to ciprofloxacin, metronidazole, and clarithromycin, respectively. However, no patients resistant to amoxicillin, tetracycline, and levofloxacin were found.

**Conclusion:**

The prevalence of *H. pylori* infection in Thailand has increased slightly. Initial regimens (triple therapy or sequential therapy or quadruple therapy) can be effective for the eradication of *H. pylori* infection, with a success rate of > 90%. For patients who failed to respond to initial triple therapy, using a longer duration of triple therapy or changing to quadruple therapy could be a good alternative. The resistance rates of amoxicillin, metronidazole, levofloxacin, and tetracycline are declining, but those of clarithromycin and ciprofloxacin are increasing.

## Graphical abstract

Initial regimens (triple therapy or sequential therapy or quadruple therapy) can be an effective regimen for the eradication of Helicobacter pylori infection, with a success rate of > 90%. For patients who failed to respond to initial triple therapy, using a longer duration of triple therapy or changing to quadruple therapy is the best choice. The resistant rates of amoxicillin, metronidazole, levofloxacin, and tetracycline are declining, but those of clarithromycin and ciprofloxacin are increasing.

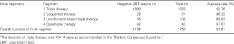

## Introduction

*Helicobacter pylori* (*H. pylori*) is a helix‐shaped, Gram‐negative, microaerophilic bacterium.

It has clinical significance as it is the root of many diseases such as gastric inflammation; atrophic gastritis; nonulcer dyspepsia; and peptic ulcer,^1^ which is the most common disease caused by *H. pylori* infection. Furthermore, *H. pylori* is a pathogen known as a definite (group I) carcinogen,^2^ and chronic infection because of this pathogen is found to be associated with gastric mucosa‐associated lymphoid tissue (MALT) lymphoma, colorectal adenomas, pancreatic cancer, and throat cancer.^1^, ^3^ According to Thailand's National Cancer Control Programmes, cancer is the most common cause of death in Thailand and is increasing. Gastric cancer is one of the top 5 most commonly diagnosed cancers in the world and is one of the top 10 causes of mortality from cancer in most Association of Southeast Asian Nations (ASEAN) countries.^4^ From this report, it can be seen that the prevalence of gastric cancer in men is gradually increasing. If *H. pylori* can be eradicated, the incidence of gastric cancer may decrease.^4^

The report of *H. pylori* infection prevalence in Thailand is quite variable or somewhat out of date. The latest *H. pylori* prevalence study in Thailand was reported in March 2018, which varied from 21 to 54%, but this study was conducted using expert opinions.^5^ In addition, the latest prevalence of 43.6% is from a systematic review, “The Global Prevalence of the *Helicobacter pylori* Infection: systematic review and Meta‐Analysis” in 2017.^6^ Although the study is a systematic review, the prevalence was assessed from only one study in Thailand, with only 179 participants, using stool antigen as the diagnostic method; hence, it is not an appropriate representation of Thai populations. However, a study with a large number of subjects was conducted from 2008–2009 and demonstrated a prevalence of 27.7%.^1^ Our study aims to review the latest prevalence of *H. pylori* infection in a large tertiary care hospital using a noninvasive method, a urea breath test (UBT), which is the best noninvasive tool for diagnosing and confirming eradication according to a Thailand consensus on *H. pylori* management in 2015.^7^ It has an excellent sensitivity of 95% and a specificity of more than 95% compared with the rapid urease test. Furthermore, this test is generally available for patients with special conditions who may be unfit to undergo the CLO test with a gastroscope. It also has the highest diagnostic accuracy for the diagnosis of *H. pylori* infection compared with other noninvasive tests (serology and stool antigen).^3^, ^6^, ^8^, ^9^

Currently, there are many recommended regimens in the guidelines for treating *H. pylori* infection. To our knowledge, the latest publication on the management of *H. pylori* in ASEAN was the Bangkok consensus report published in 2018.^4^ It suggested that the first‐line regimen should be based on antibiotic resistance patterns in each area, while the second‐line regimen should not contain the same drug as the first‐line regimen, and antibiotic susceptibility testing should be performed when there are more than two treatment failures.^4^, ^5^

Nowadays, medical professions worldwide encounter the increasing trend of drug‐resistant bacteria. This problem alone can immensely affect the success rate of the treatment regimen for *H. pylori* infection because each regimen consists of different kinds of antibiotics used to treat other medical illnesses as well. We conducted this study to explore the present‐day success rate of each regimen, to compare with recommendations from Thailand and ASEAN, and perhaps suggest a suitable regimen from real‐world experience. In addition, we aim to identify antibiotic resistance patterns in the current situation and to suggest a regimen to guide physicians in treating the patient with *H. pylori* infection resistant to antibiotics.

## Methods

### *Data collection*

This is a descriptive retrospective study. Any patients who underwent UBT in the Gastroenterology Unit, Faculty of Medicine, Chulalongkorn University during the years 2013–2017 were all included in the study. Data from those patients were collected, including indications for UBT, antibiotic regimen used, UBT results, and prior antibiotics used in each patient. People who had incomplete treatment data or missing data were excluded from the study. The patients were divided into a diagnostic group and a posttreatment group. The “diagnostic” group includes patients with dyspeptic symptoms who underwent UBT for the diagnosis of *H. pylori* infection, while the “posttreatment” group includes patients who were previously diagnosed with *H. pylori* infection and underwent UBT as a follow‐up test after the treatment.

**Figure 1:**
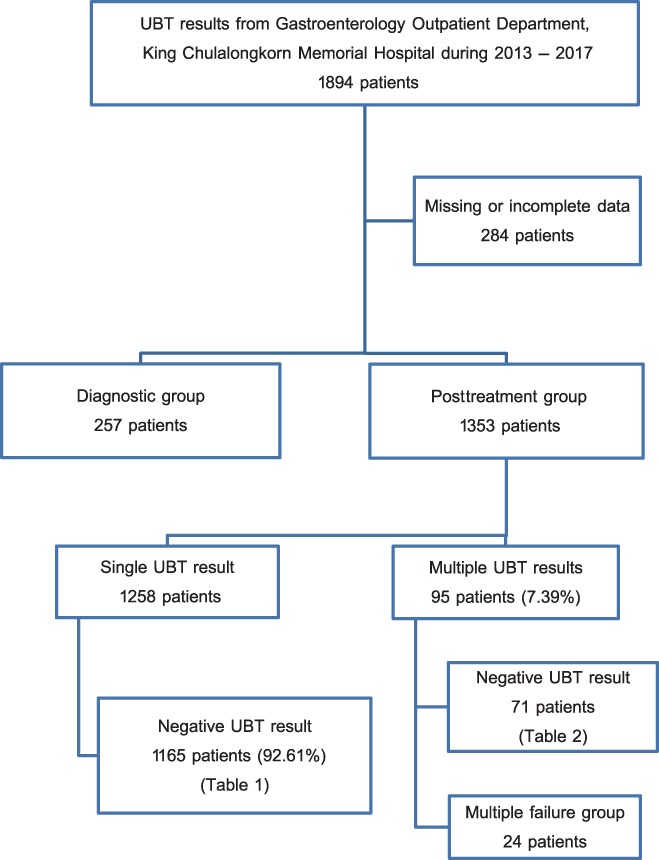
Flow diagram of the study. UBT, urea breath test.

Patients with a positive UBT result in the “posttreatment” group indicate unsuccessful treatment and were categorized as the “treatment failure” group. Some of these patients underwent *H. pylori* culture process. We collected the results of *H. pylori* culture to explore the drug‐resistant situation. Furthermore, several patients underwent UBT more than once during the period of 5 years, and their results will be discussed later. All data are shown in Fig. 1.

### *UBT method*

All patients underwent the “^13^C‐Urea Breath Test” from Otsuka Pharmaceutical Co., Ltd. Tokyo, Japan. This test comes with two sterile sealed plastic bags and a UBIT tablet that contains 100 mg of urea (^13^C). Patients must discontinue proton pump inhibitors for at least 14 days and must stop antibiotic use for at least 28 days before undergoing UBT. After fasting for at least 6 h prior to the test, patients sat in the upright position and blew into the pre‐urea test sealed bag until it fully expanded. After that, the patient swallowed the UBIT tablet, lay on the left side for 5 min, and changed positions to sitting upright for another 15 min. The next step was blowing into the second bag marked posturea test; both bags were placed in a mass spectrum machine for processing. The results can be obtained in 2 min. The mechanism behind the detection of *H. pylori* infection is as follows: When a patient swallows an uncommon isotope—in this case a nonradioactive C‐13—if *H. pylori* is present, the urease enzyme from *H. pylori* will split the urea, and an isotope‐labeled carbon dioxide will be detected in the exhaled breath. The difference in amounts between 12 C and 13 C will be calculated, and a positive result cut‐off level is observed to be ≥2.5%. The test's sensitivity is 97.7% and specificity is 98.4%.

### *H. pylori culture method*

Three specimens from antrum were transported in sterile, screw‐capped tubes with a cysteine medium inside. One specimen was used for rapid urease test, and the others were used for cultures and drug susceptibility tests. The specimen used for culture were inoculated on both nonselective horse blood agar (HBA) plate and selective HBA plate containing vancomycin and amphotericin. They were incubated at 37°C in microaerophilic conditions for 72 h, and the results were recorded consecutively for 2 weeks. *H. pylori* were identified by colony morphology; Gram stain reaction; and catalase, urease, and oxidase reactions. The specimens from successful culture plates were placed on a fresh HBA plate and were then incubated for 72 h. When the number of bacteria was enough to achieve 2 McFarland turbidity unit, an *H. pylori* specimen from Columbia broth was placed on HBA plates. Epsilometer tests (*E*‐test; AB Biodisk, Solna, Sweden) were used to test antibiotic susceptibility, with antibiotics such as amoxicillin, clarithromycin, metronidazole, and tetracycline.

The minimum inhibitory concentration (MIC) was interpreted by readings at the intersection point between the inhibitory zone and the tester after they were incubated together for 72 h. MIC interpretation criteria from the European Committee on Antimicrobial Susceptibility Testing (EUCAST) were used for amoxicillin (>0.125 μg/mL), metronidazole (>8 μg/mL), and tetracycline (>1 μg/mL), while MIC interpretation criteria for clarithromycin (≥1 μg/mL) were obtained from the Clinical and Laboratory Standards Institute (CLSI).^10^, ^11^

## Results

The UBT records from the patients with dyspeptic symptoms included 1894 patients during the years 2013–2017. A total of 284 patients were excluded from the study due to incomplete or missing data. The remaining results were categorized into the “diagnostic” group of 257 patients and the “posttreatment” group of 1353 patients (Fig. 1).

In the “diagnostic” group, 73 of 257 patients had positive results. Hence, the prevalence of *H. pylori* infection diagnosed from the UBT is 28.4%. In the “posttreatment” group, 1258 of 1353 patients had a single UBT result after the initial treatment for *H. pylori* infection. Among them, 1165 patients had negative results, which means that 92.61% of patients were successfully treated for *H. pylori* by initial treatment. The success rate of each regimen is shown in Table 1.

**Table 1:** The success rate of initial regimens

Initial regimens	Regimen	Negative UBT results (*n*)	Total (*n*)	Success rate (%)
	1 Triple therapy†	1000	1078	92.76
	2 Sequential therapy	28	31	90.32
	3 Levofloxacin‐based triple therapy	95	106	89.62
	4 Quadruple therapy	42	43	97.67
Overall success of initial regimen		1165	1258	92.61

† The duration of triple therapy was 10–14 days as recommended in the Thailand Consensus Guideline.^7^

UBT, urea breath test.

In the “posttreatment” group, 95 patients had undergone UBT more than once, and the first UBT result was positive, which means that *H. pylori* was not eradicated by the initial regimen. We call these patients the “initial treatment failure” group. This group received various treatments; 84 patients who failed to respond to the initial triple therapy regimen were prescribed an extended duration of triple therapy, sequential therapy, levofloxacin‐based triple therapy, and quadruple therapy. All data are presented in Table 2. Patients who were prescribed an extended duration of triple therapy achieved an 88.24% success rate of *H. pylori* eradication. The success rate in patients who were treated with sequential therapy was 53.85%. The efficacy of levofloxacin‐based triple therapy was 55.56%. Finally, quadruple therapy showed a success rate of 69.44%. Of nine patients who failed to respond to levofloxacin‐based triple therapy, three received sequential therapy; 66.67% responded to this regimen, and 66.67% were successfully treated with quadruple therapy. There is one patient who failed to respond to initial sequential therapy but responded to levofloxacin‐base triple therapy, and there is also one patient who failed to respond to quadruple therapy but successfully beat *H. pylori* infection through levofloxacin‐based triple therapy.

**Table 2:** Regimens used after failure of initial regimens

Initial regimens	Choices of second regimens	Total (*n*)	Success rate (%)
Triple therapy (*n* = 84)	Triple therapy†	17	88.24
	Sequential therapy	13	53.85
	Levofloxacin‐based triple therapy	18	55.56
	Quadruple therapy	36	69.44
Sequential therapy (*n* = 1)	Levofloxacin‐based triple therapy	1	100
Levofloxacin‐based triple therapy (*n* = 9)	Sequential therapy	3	66.67
	Quadruple therapy	6	66.67
Quadruple therapy (*n* = 1)	Levofloxacin‐based triple therapy	1	100
Overall success of the second attempt antibiotics regimen		95	68.42

† Triple therapy was used in different antibiotics or at a higher dose or longer duration.

Of 95 patients from the “initial treatment failure” group, 24 received multiple treatment regimens but still had positive UBT result and were categorized into the “multiple failure group.” All data are shown in Table 3. After using UBT as a guide, only two patients' treatment results were found, which is still positive. One patient received triple therapy, levofloxacin, and quadruple therapy, and the other received triple therapy followed by quadruple therapy thrice. After failure to respond to the stated treatment regimens, both were prescribed levofloxacin.

**Table 3:** Antibiotic regimens used in “multiple failure” patient group

First regimen after initial treatment failure	Second regimen after initial treatment failure	Number of patients (*n* = 21)
Triple therapy	Quadruple	9
Triple therapy	Sequential	4
Triple therapy	Levofloxacin	4
Levofloxacin	Quadruple	2
Levofloxacin	Sequential	1
Triple	Triple	1

First regimen after initial treatment failure	Second regimen after initial treatment failure	Third regimen after initial treatment failure	Fourth regimen after initial treatment failure	Number of patients (*n* = 3)
Triple	Levofloxacin	Quadruple	No data	1
Triple	Sequential	Levofloxacin	Sequential	1
Triple	Quadruple	Quadruple	Quadruple	1

Combining 93 patients with a positive result from the single UBT result group and 95 patients from the “initial treatment failure” group, we obtained a total of 188 patients in whom *H. pylori* eradication failed. Among them, 40 instances of *H. pylori* culture were conducted in 28 patients. Of the 40 instances, 16 (40%) culture results were positive. According to EUCAST and CLSI criteria, there was no report for amoxicillin, tetracycline, and levofloxacin resistance. However, there were six patients who demonstrated resistance to ciprofloxacin (21.43%) (five females and one male), four patients (14.29%) (three females and one male) who were resistant to metronidazole, and three patients who were resistant to clarithromycin (10.71%) (two females and one male). The MIC result is shown in Table 4. Moreover, the male patient who resisted metronidazole, clarithromycin, and ciprofloxacin was the same patient.

**Table 4:** Antibiotic resistance and MIC (μg/mL) results (28 patients)

Antibiotics	MIC (μg/mL)	Resistant rate (%)
Amoxicillin	≥0.5	0
Metronidazole	>8	14.29
Tetracycline	>8	0
Clarithromycin	>1	10.71
Levofloxacin	>1	0
Ciprofloxacin	>1	21.43
Multiple drugs resistant	—	3.57

MIC, minimum inhibitory concentration.

## Discussion

According to the latest study, the estimated global *H. pylori* infection in 2017 was approximately 4.4 billion people.^6^ The latest prevalence rates in patients who underwent esophagogastroduodenoscopy (EGD) in King Chulalongkorn Memorial Hospital between 2008 and 2009 was 27.7%.^12^ Four years later, the prevalence of *H. pylori* infection from our study was 28.4%, and 68.5% of the study population are male. Similar to the previous study, male patients still predominate with regard to *H. pylori* infection. This may be because men are more likely to smoke and drink alcohol than women.

The prevalence of *H. pylori* infection has increased compared to the previous study.^12^ Factors affecting *H. pylori* infection prevalence are different populations and different methods. The previous study was conducted using either symptomatic or asymptomatic screening patients who underwent EGD.^12^ Meanwhile, the populations in our study are all symptomatic patients. Therefore, a higher prevalence is expected. In addition, our populations are more diverse because we also recruited patients who have contraindications for EGD, for example, coagulation defect and hemodynamic instability.

Comparing between the first‐line regimens, using triple therapy is as effective as sequential therapy, while in the second‐line regimen, Bismuth‐based quadruple therapy is more effective than levofloxacin‐based triple therapy. We can conclude that triple therapy is still the most effective regimen. This may be described by the complexity of sequential therapy and its side effects that affects patients’ compliance.

From our study, patients who failed to respond to first treatment by triple therapy received quadruple therapy the most (eradication rate 69.44%), levofloxacin‐based triple therapy (eradication rate 55.56%), triple therapy again (eradication rate 88.23%), and sequential therapy (eradiation rate 53.85%). We noticed that, if patients fail to respond to triple therapy as the first regimen, using triple therapy for a longer duration is still the best choice. However, Thailand Consensus guideline^7^ recommended that quadruple therapy, which is a second‐line treatment, can also be used. Our study found that the success rate of using quadruple therapy after failure of initial triple therapy is 69.44%, which is still unacceptable in clinical practice. Further studies with more patients are required to prove if this this regimen is effective in real life.^7^

There was a patient who was prescribed levofloxacin‐based triple therapy after failure of initial treatment by sequential therapy and was successful in the eradication of *H. pylori*. Besides, the patient with a history of failure to respond to initial treatment by quadruple therapy can be successfully treated by levofloxacin‐based quadruple therapy. Nine patients who failed to respond to levofloxacin‐based triple therapy were divided into two groups. The first group was prescribed sequential therapy, and two of three patients successfully responded. Another group was prescribed with quadruple therapy, and two of four patients successfully responded. Due to a small number of patients, further studies should be conducted to identify the effectiveness of treatment by nonfirst‐line regimens as the initial treatment for patients with *H. pylori* infection with contraindication to the use of first‐line regimen, for example, drug allergy or severe side effects.

According to the Thailand Consensus on *H. pylori* management in 2015, an antibiotic susceptibility test will be performed in patients who failed to respond to the treatment. Of 188 patients who failed to respond, 28 were tested for *H. pylori* culture and drug susceptibility. Of 40 culture plates, 60% turned out to be negative, although the UBT results were positive. This may be due to a lack of proper temperature control when transporting the media in the hot weather in Thailand, a small number of organisms, or a biopsy sample from an uncommon site of *H. pylori* infection.

From the drug susceptibility test, no drug resistance for amoxicillin, tetracycline, and levofloxacin was found. Meanwhile, compared to the published study in 2014, the resistance rates of amoxicillin, tetracycline, and levofloxacin were 5.2, 1.7, and 7.2%, respectively. The resistance rates of ciprofloxacin, metronidazole, and clarithromycin are 15, 10, and 7.5%, respectively, compared to 7.7, 36, and 3.7% from the same study. The resistance rate of amoxicillin decreased to zero, perhaps because of the current rational drug use trend and because physicians are more likely to either prescribe no antibiotic for upper respiratory tract infection or escalate antibiotic use to a higher‐potency group. The resistance rate of levofloxacin declined dramatically, possibly because it was reserved for use in only severe pulmonary infection and tuberculosis. The ciprofloxacin resistance rate increases because it is the most common drug used for urinary tract and gastrointestinal tract infections in Thailand. From our study, six of seven patients who are resistant to ciprofloxacin are female. This can be explained by the general knowledge that women have a short urethra and are prone to experience urinary tract infections more than men. Although metronidazole is the most common antibiotic used in Thailand for treating gastrointestinal tract infections, parasitic infections, and other surgical conditions, the metronidazole resistance rate has drastically decreased. We presume that physicians may choose alternative antibiotics that have the same treatment spectrum but fewer side effects than metronidazole. The resistance rate of clarithromycin has increased, perhaps because of the considerably increasing prescription of clarithromycin for empirical respiratory tract infections. The limitation of this study is that it is a retrospective study; as a result, we could not collect all data, and the culture rate was too low. Moreover, the eradication rate is influenced by factors other than resistance, such as compliance. Hopefully, a prospective study will be conducted in the future using a newer technique of culture.

In conclusion, the prevalence of *H. pylori* infection in dyspeptic patients in Thailand is still high (28.4%) and demonstrates male predominance. Initial regimens (triple therapy or sequential therapy or quadruple therapy) can be an effective regimen for the eradication of *H. pylori* infection, with a success rate of > 90%. The recommended regimen after failure to respond to initial therapy is a longer duration and higher doses of triple therapy or quadruple therapy. The antibiotic resistance trend has shifted to less resistance to amoxicillin, metronidazole, levofloxacin, and tetracycline. Conversely, the resistance rates of in clarithromycin and ciprofloxacin have increased.
